# Systematic review of studies investigating ventilator associated pneumonia diagnostics in intensive care

**DOI:** 10.1186/s12890-021-01560-0

**Published:** 2021-06-09

**Authors:** Basem Al-Omari, Peter McMeekin, A. Joy Allen, Ahsan R. Akram, Sara Graziadio, Jana Suklan, William S. Jones, B. Clare Lendrem, Amanda Winter, Milo Cullinan, Joanne Gray, Kevin Dhaliwal, Timothy S. Walsh, Thomas H. Craven

**Affiliations:** 1 College of Medicine and Health Sciences, Khalifa University, PO Box 127788, Abu Dhabi, UAE; 2 School of Health and Life Science, University of Northumbria, Newcastle upon Tyne, UK; 3 NIHR Newcastle In Vitro Diagnostics Co-operative, Translational and Clinical Research Institute, Newcastle University, Newcastle upon Tyne, UK; 4 Translational Healthcare Technologies Group, Centre for Inflammation Research, Queen’s Medical Research Institute, University of Edinburgh, Edinburgh, UK; 5 York Health Economics Consortium, Enterprise House, Innovation Way, University of York, York, UK; 6 NIHR Newcastle In Vitro Diagnostics Co-operative, The Newcastle Upon Tyne Hospitals NHS Foundation Trust, Newcastle upon Tyne, UK; 7 Laboratory Medicine, Newcastle-Upon-Tyne Hospitals Foundation Trust, Newcastle upon Tyne, UK; 8 Edinburgh Critical Care Research Group, University of Edinburgh, Edinburgh, UK

**Keywords:** Ventilator-associated pneumonia, Diagnostics, Intensive care, Critical care

## Abstract

**Background:**

Ventilator-associated pneumonia (VAP) is an important diagnosis in critical care. VAP research is complicated by the lack of agreed diagnostic criteria and reference standard test criteria. Our aim was to review which reference standard tests are used to evaluate novel index tests for suspected VAP.

**Methods:**

We conducted a comprehensive search using electronic databases and hand reference checks. The Cochrane Library, MEDLINE, CINHAL, EMBASE, and web of science were searched from 2008 until November 2018. All terms related to VAP diagnostics in the intensive treatment unit were used to conduct the search. We adopted a checklist from the critical appraisal skills programme checklist for diagnostic studies to assess the quality of the included studies.

**Results:**

We identified 2441 records, of which 178 were selected for full-text review. Following methodological examination and quality assessment, 44 studies were included in narrative data synthesis. Thirty-two (72.7%) studies utilised a sole microbiological reference standard; the remaining 12 studies utilised a composite reference standard, nine of which included a mandatory microbiological criterion. Histopathological criteria were optional in four studies but mandatory in none.

**Conclusions:**

Nearly all reference standards for VAP used in diagnostic test research required some microbiological confirmation of infection, with BAL culture being the most common reference standard used.

**Supplementary Information:**

The online version contains supplementary material available at 10.1186/s12890-021-01560-0.

## Take home message

1. This comprehensive systematic review assesses the reference standard tests used to evaluate novel index tests for suspected VAP in ICU over 10 years period (2008 until 2018) and included high-quality studies with low risk of bias.
2. BAL culture is the most common reference standard used for VAP diagnostic in ICU and almost all reference standards required some microbiological confirmation of infection.

## Background

Ventilator-associated pneumonia (VAP) refers to inflammation of the lung parenchyma caused by infectious agents acquired specifically while receiving invasive mechanical ventilation [1, 2]. VAP is a preventable nosocomial complication which potentially contributes to avoidable mortality and morbidity [3, 4]. Therefore, it is considered a clinically and epidemiologically important measure of the quality of care [5, 6]. It contributes to additional resource consumption, adding time and expense to an intensive care stay, accounting for a large proportion of all antibiotic prescriptions [7]. VAP is considered to be responsible for an additional cost of approximately $40,000 per episode in the US [8, 9] and around £9000 in the UK [10]. The contribution of an episode of VAP to mortality is difficult to definitively ascertain because of the high number and severity of confounders amongst the at-risk population [4, 11–13]. This attributable mortality has been reported from high to neutral or near-neutral [11–13].

Throughout recent decades investigators have not adopted a fixed set of criteria or a fixed definition for VAP [14]. This lack of a reference standard has led to an inability to make comparisons across study sets and uncertainty about VAP incidence [15]. The incidence of VAP varies widely in different studies depending on the diagnostic criteria used, type of intensive therapy unit (ITU), and patient population [16, 17].

Existing literature reports that the incidence of VAP varies widely between 4.0% and 28.8% of the at-risk population [8, 18–24], with an event rate between 1.4 and 16.5 per 1000 ventilator days [1, 25–27]. As VAP rates have become an important quality indicator, the Centre for Disease Control and the European Centre for Disease Control use their own precise case definitions to identify VAP events [28, 29]. Both definitions return similar VAP rates making them adequate for surveillance purposes and benchmarking of critical care units internationally [1]. However, due to the lack of concordance between these two definitions, they do not make ideal reference standards [1, 28], and further highlight the difficulty in achieving consensus in diagnosing VAP. Microbiological samples, especially quantitative culture of bronchoalveolar lavage (BAL), are considered to be integral to the diagnosis of VAP [30, 31]. However, a systematic review of diagnostic methods in 2008 found that microbiological methods did not contribute to the accuracy of diagnosis over clinical criteria and all respiratory sampling methods were equivalent [32]. The continuing lack of an agreed reference standard hampers research into novel diagnostic methods. The aim of this review was to identify what reference standards have been used in diagnostic evaluation research for VAP.

## Methods

The protocol for this review was published in PROSPERO (International Prospective Register of Systematic Reviews) under registration CRD42019125449 [33].

### Search strategy

A comprehensive search strategy was developed by one of the authors (BA). The Cochrane Library, PubMed (MEDLINE), CINHAL, EMBASE, and web of science were electronically searched from January 2008 until November 2018. We limited our search to studies published after 2008 following a comprehensive systematic review of diagnostic methods [32]. Medical Subject Headings (MeSH) and search terms were used to interrogate the databases. The 3 concepts used for the searches were VAP, diagnostics, and ITU (for search terms see Additional file 1). No restriction on publication language was applied. In addition, electronic searching of Google and hand searching through an examination of the reference list of the published articles were also used to identify additional publications (an example of MEDLINE search is provided in Additional file 1).

### Review strategy

All records were independently reviewed by the lead author (BA) and another author (PM or JG) and disagreement was resolved by a third independent adjudicator (PM or JG). Initially, titles and abstracts review of all records, then full-text reviews were conducted against the inclusion/exclusion criteria. Studies included in the review fulfilled the following criteria: (1) adult ventilated patients of any gender, (2) ITU settings, (3) suspected VAP as defined in this study (after 48 h on the ventilator), (4) focused on the diagnostic procedures of VAP (clinical markers, biomarkers, chest x-ray, chest ultrasound (U/S), lung biopsy, BAL and mini-BAL, protected specimen brush (PSB), blind PSB, Endotracheal Aspirate (ETA)). Studies were excluded from the review if they: (1) were animal studies, (2) included patients under the age of 18 years old, (3) focused on the surveillance of VAP, (4) compared the diagnosis of VAP against another illness diagnostic, (5) were feasibility studies, (6) included participants who were already diagnosed with VAP, (7) investigated VAP treatment effectiveness by monitoring biomarkers or other diagnostics, (8) evaluated risk factors to predict VAP, (9) were procedures used to predict the mortality in VAP, (10) were case-controlled studies. All papers that passed the full-text review and those that had some diagnostic technical terms were examined by an ITU clinician (THC) to confirm their clinical relevance to the research question.

### Quality assessment and data extraction

A team of 12 reviewers (systematic reviewers, clinicians, methodologists, health economists) from the University of Northumbria, Newcastle University, The Newcastle Upon Tyne Hospitals NHS Foundation Trust, and the University of Edinburgh were involved in the quality assessment and data extraction process. All included papers were quality assessed and the data were extracted by two authors independently. Any disagreement was discussed between both reviewers in the first instance. The further disagreement was resolved by a third reviewer. The quality assessment scoring checklist was adopted from the Critical Appraisal Skills Programme (CASP) checklist for diagnostic studies [34], which is one of the well-recognised methodological quality or risk of bias assessment tools for primary and secondary medical studies [35, 36] and has been used to assess the quality of diagnostic studies in systematic reviews [37–39]. The quality assessment scoring checklist contains 8 questions from the overall 12 questions in the CASP checklist. Questions from section C in the CASP for diagnostics checklist “*will the results help locally?*” were not included in our scoring as the main aim of the review was not related to the local application of the diagnostic procedures. Studies were assigned a score of ‘1’ for each item of the checklist if they were considered to meet the aspect of this item and ‘0’ if not. A total score for each study was calculated by summing the item scores. The maximum possible final score was 8. Any study that scored ‘0’ for the first or the second question or scored less than ‘5’ out of 8 in total was excluded. According to CASP guide for diagnostic studies, if the answer to question 1 or 2 while critically appraising a study was “no”, then it is not worth continuing. That leaves 6 questions out of the total 8 we used in our quality assessment. Taking in consideration that these questions are equally as important but less important than the first 2 question, we determined that a study must fulfil the quality of at least half of these 6 points (score 3 out of 6) to be consider for the review. Therefore, this threshold was derived through reviewer consensus that studies scoring less than 5 out of 8 were not of sufficient quality to adequately address the research question.

A standardised data extraction form was developed by three authors (AJA, BA, THC) and reviewed by all authors (for quality assessment and data extraction form see Additional file 2). We recorded and present study country of origin, study size, male: female ratio or enrolled participants, index test(s) under investigation, reference standard used to define VAP, and test characteristics. Although test characteristics for the index test are not relevant to the aims of this review, we present them herein because several of the index tests are also used as reference standards. Test characteristics are taken directly from the studies or calculated using data contained within the studies. Where multiple test characteristics are presented in the original paper, we selected those highlighted by the original authors or those which reflect the comparison best, or those which indicate the best performance. Where BAL was conducted, we recorded the details of the lavage procedure.

A narrative data synthesis approach was used to report the results from reviewed studies. Due to the large variation in practice, processes, and reference standards, a meta-analysis of diagnostic accuracy was not conducted.

## Results

### Studies identified

**Fig. 1:**
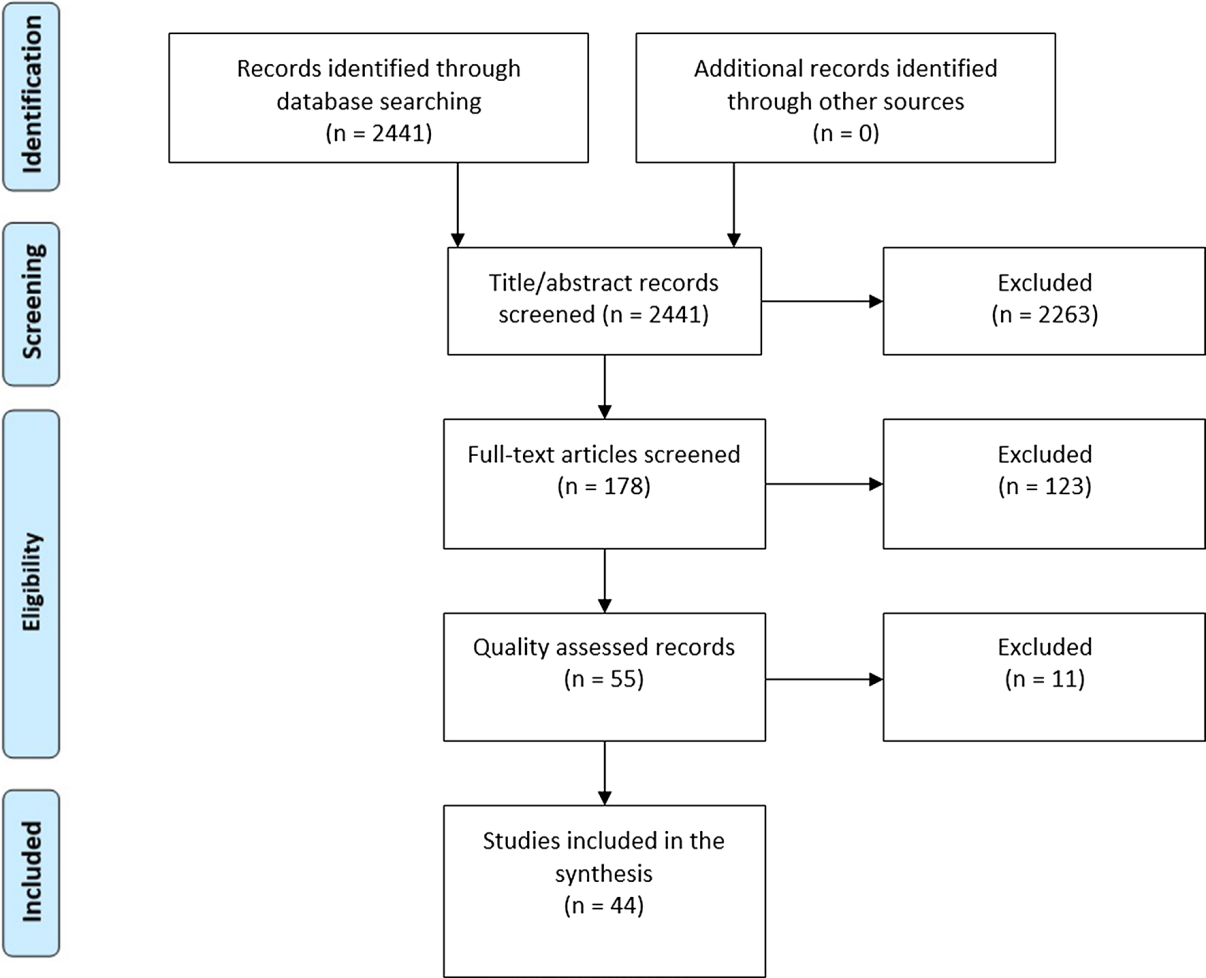
PRISMA systematic review flow chart. Adapted from: Moher et al. [96]

The searches identified a total of 2441 articles. Records that were not published in English were translated to English using Google translator. 2263 articles were excluded on the basis of title and abstract and a further 123 on the basis of full-text screening were excluded as not clinically relevant to the inclusion criteria or meeting at least one of the exclusion criteria, leaving 55 articles for quality assessment (see Fig. 1 for PRISMA flow chart).

### Quality assessment and data extraction

Of the 55 studies examined in the quality assessment stage, 11 studies were excluded due to either scored ‘0’ for the first or the second question or scored less than ‘5’ out of’8’ in total score, leaving 44 studies included in this review [40–83]. All scored were agreed by at least two reviewers and reviewed by the principal investigator (PI). The lowest score assigned to any included study was ‘5’ out of ‘8’. Three studies scored 8/8, 24 studies scored 7/8, 11 studies scored 6/8, and six studies scored 5/8 (see Table 1).

**Table 1:**
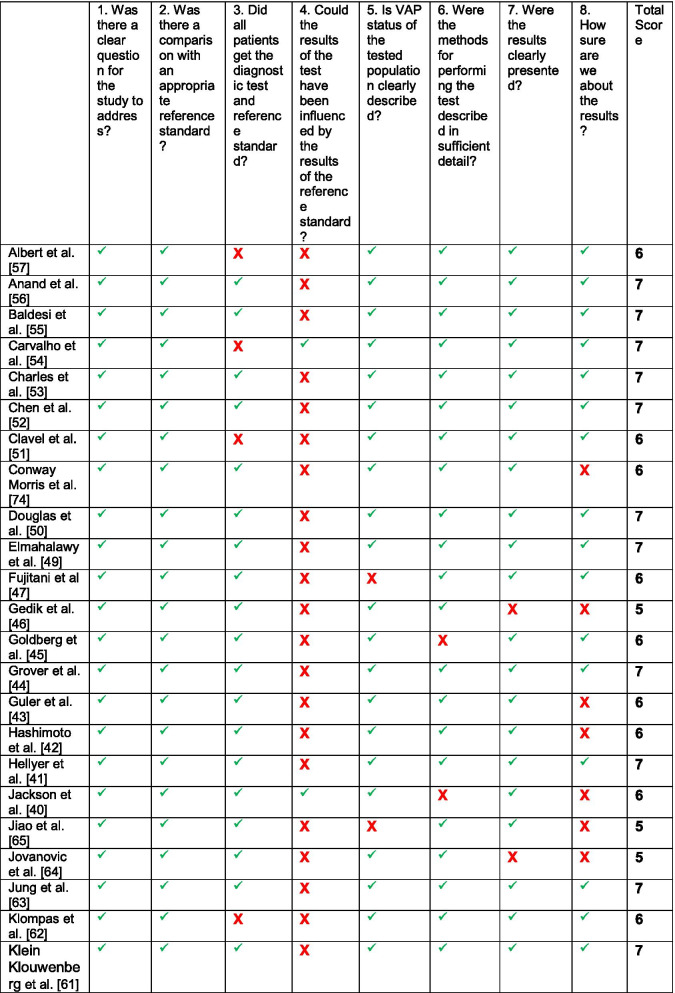
Quality assessment checklist scores

*green tick* = acceptable quality, *red box* = unacceptable quality

As expected, all papers suffered from bias in their accuracy estimates of the index test from the use of an imperfect reference standard comparison, a well-known issue with comparative diagnostic accuracy studies [84]. The results of the quality assessment reviews conducted using the form adopted from CASP diagnostic study checklist showed that five papers [51, 54, 57, 62, 82] suffered from verification bias: not all patients received testing by both the index and the reference standard. In 38 papers [41–47, 49–53, 55–59, 61–68, 70, 71, 73–82], the results of the index test could have been influenced by the reference standard result. This means that there was no evidence blinding or the tests being performed independently. The VAP status for all participants in the study was not clearly defined in two papers [47, 65]. The methodology description was not described in detail in three papers [40, 45, 82] and the results of the study were not clearly presented in five papers [46, 64, 67, 69, 75]. There was a lack of certainty regarding the results of the study on 11 occasions [40, 42, 43, 46, 58, 64, 65, 67, 68, 74, 75]. CASP diagnostic study checklist guide was followed in assessing all points.

The disagreement was solved through discussion between both reviewers on 20 papers, and adjudication of a third reviewer in one paper. Forty-three of the studies were cohort studies (38 of which were prospective studies) and one was a secondary analysis of data from a randomised controlled trial (RCT) [57]. All studies were published between 2008 and 2018 with a wide geographical spread; four studies conducted in the UK [41, 44, 72, 74], eight in the USA [40, 45, 47, 50, 56, 62, 67, 75], seven in France [51, 53, 55, 63, 70, 73, 78], five in Netherland [48, 61, 66, 77, 79], three each in both China [52, 65, 68] and Turkey [43, 46, 82], two each in Egypt [49, 76], Brazil [54, 81], and Italy [71, 83], and eight in other countries [42, 57–60, 64, 69, 80]. The median sample size was 180 recruited participants, with the 21 being the lowest [58] and 2080 being the highest number of participants [61] (see Tables 2, 3).

**Table 2:** Studies comparing index test to a microbiological reference standard

References	Study population				Index test	Reference test	Results (95% confidence interval where available)
	Country	*n* =	Mean age	M:F ratio			
Albert et al. [57]	Canada	705	58.9	2.9	Gram stain of either ETA or BAL	same sample (ETA or BAL) positive culture result for pathogenic bacteria any quantity	Sens = 74% Spec = 72% κ = 0.36 (0.31–0.40)
Anand et al. [56]	USA	105	51.5	1.4	sTREM-1 ELISA in BAL	BAL culture > 10^3^ CFU/mL	Sens = 42.1% Spec = 75.6%
Baldesi et al. [55]	France	127	62.5	2.3	BAL culture > 10^4^ CFU/mL (uncorrected for dilution factor)	BAL culture > 10^4^ CFU/mL (corrected for dilution factor using plasma/BAL urea)	Sens = 97.7% Spec = 98%
Carvalho et al. [54]	Brazil	22	59.41		comparison of three sampling techniques (ETA, miniBAL, and BAL)	No mention which is considered index test or reference standard	Sens = NR Spec = NR
Clavel et al. [51]	France	120	65	3.1	qPCR from BAL equivalent to > 10^4^ CFU/mL and qPCR from ETA equivalent to > 10^6^ CFU/mL	BAL culture > 10^4^ CFU/mL or ETA culture > 10^6^ CFU/mL	*qPCR from BAL* Sens = 89.2% Spec = 97.1% *qPCR from ETA* Sens = 71.8% Spec = 96.6%
Conway Morris et al. [74]	UK	72	57	1.5	BAL IL-1β and IL-8	BAL culture > 10^4^ CFU/mL	Sens = 94% Spec = 64%
Douglas et al. [50]	USA	33	55	N/R	Mini BAL automated microscopy	Mini BAL culture > 10^4^ CFU/mL	Sens = 100% Spec = 97%
Elmahalawy et al. [49]	Egypt	40	45.4	3.0	Pentraxin 3 ELISA in BAL	BAL culture > 10^4^ CFU/mL	Sens = 96.7% Spec = 100% AUROCC = 0.966 (0.985 to 1)
Fujitani et al. [47]	USA	256	57.5	2.1	ETA culture	Mini BAL culture > 10^4^ CFU/mL	Sens = 65.4% Spec = 56.1% κ = 0.22 (0.17–0.27)
Gedik et al. [46]	Turkey	31	56	1.4	ETA culture > 10^5^ CFU/mL	Mini BAL culture > 10^4^ CFU/mL	κ = 0.804, p = 0.005
Goldberg et al. [45]	USA	229	49	2.0	Gram stain of BAL	BAL culture > 10^5^ CFU/mL	Sens = 90% Spec = 67%
Guler et al. [43]	Turkey	50	52	2.1	Modified CPIS	BAL culture > 10^4^ CFU/mL	*CPIS > 7* Sens = 80% Spec = 17% *CPIS > 6* Sens = 76% Spec = 15% *CPIS ≥ 6* Sens = 80.7% Spec = 16.6% *CPIS ≥ 5* Sens = 80% Spec = 10%
Hashimoto et al. [42]	Japan	51	41	1.4	Semi-quantitative Gram stain or culture of ETA or BAL (not otherwise specified)	ETA culture > 10^6^ CFU/mL	*Gram stain* Sens = 95% Spec = 61% κ = 0.59 (0.45–0.72) *Culture* Sens = 96% Spec = 40% κ = 0.4 (0.25–0.54)
Hellyer et al. [41]	UK	150	56.3	2.9	BAL IL-1β and IL-8	BAL culture > 10^4^ CFU/mL	Sens = 100% Spec = 44.3%
Jackson et al. [40]	USA	73	52	3.5	BAL—unilateral	BAL—bilateral	Sens = 73% Spec = 82%
Jiao et al. [65]	China	92	48	1.0	Serum PCT Serum IL-6 Serum CRP	BAL culture > 10^4^ CFU/mL	*PCT* Sens = 91% Spec = 71% *IL-6* Sens = 58% Spec = 64% *CRP* Sens = 52% Spec = 74%
Jung et al. [63]	France	57	61	2.2	BAL Procalcitonin	BAL culture > 10^4^ CFU/mL	Sens = 94% Spec = 75%
Kneidinger et al. [60]	Austria	132	62	2.1	BAL culture > 10^4^ CFU/mL after 24 h storage at 4 °C and after storage at − 80 °C	BAL culture > 10^4^ CFU/mL after immediate culture	*Immediate culture vs delayed culture (4 °C)* Sens = 96.5% Spec = 100% *Immediate culture vs delayed culture (*− *80 °C)* Sens = 50.4% Spec = 100%
Kwon et al. [59]	Korea	64	65	2.6	qPCR of BAL fluid or bronchial washings	BAL culture > 10^4^ CFU/mL or bronchial washing > 10^5^ CFU/mL	Sens = 88.9% Spec = 88.9%
Leo et al. [58]	Mexico	21	42	2.0	mini BAL (using cut NG tube) > 10^4^ CFU/mL	BAL culture > 10^4^ CFU/mL	Sens = 92.8% Spec = 85%
Linssen et al. [67]	USA	117	N/R	N/R	BAL PCT and CRP	BAL culture > 10^4^ CFU/mL or > 2% cells with intracellular organisms	*PCT* AUCROCC = 0.448 *CRP* AUCROCC = 0.477
Linssen et al. [75]	USA	282	60.3	2.1	Various BAL cytological parameters including total cell count, alveolar macrophages, lymphocytes, neutrophils, eosinophils, mast cells, infected cells; in isolation or in combination	BAL culture > 10^4^ CFU/mL	*AUROCC for various cytological parameters* Total cell count = 0.647 Alveolar macrophages = 0.313 Lymphocytes = 0.381 Neutrophils = 0.705 Eosinophils = 0.589 Mast cells = 0.557 Infected cells (%) = 0.904 *AUROCC for combinations of cytological parameters* Infected cells (%) + PMNs (%) = 0.892 Infected cells (%) + Total cell count = 0.890
Luna et al. [69]	Argentina	283	71.3	1.1	ETA culture > 10^3^ CFU/mL	BAL culture > 10^4^ CFU/mL	Sens = NR Spec = NR
Luyt et al. [70]	France	41	60	2.4	serum PCT 1^st^ day of clinical suspicion of VAP	BAL culture > 10^4^ CFU/mL	Sens = 72% Spec = 24%
Mongodi et al. [73]	France	99	66	3.7	Vplus-Eagram (ventilator-associated pneumonia lung ultrasound score and gram stain of ETA)	BAL culture > 10^4^ CFU/mL	Sens = 81% Spec = 41%
Oudhuis et al. [66]	Netherlands	207	62	1.7	ETA culture > 10^5^ CFU/mL	BAL culture > 10^4^ CFU/mL or > 2% cells with intracellular organisms	Sens = 65% Spec = 48%
Refaat et al. [76]	Egypt	66	NR	NR	BAL composite biomarker (CRP, PCT, neutrophils, MIF, sTREM-1, suPAR)	BAL culture > 10 CFU/mL	Sens = 76% Spec = 62%
Schnabel et al. [48]	Netherlands	100	61.3	2.3	VOC in exhaled breath	BAL culture > 10^4^ CFU/mL or > 2% cells with intracellular organisms	Sens = 75.8% Spec = 73.0%
Scholte et al. [77]	Netherlands	311	NR	NR	ETA Gram stain ETA Culture BALF Gram stain	BAL culture > 10^4^ CFU/mL or > 2% cells with intracellular organisms	*ETA Gram stain* Sens = 65% Spec = 72% *ETA Culture (any threshold)* Sens = 82% Spec = 42% *BALF Gram stain (> 0)* Sens = 82% Spec = 66%
Vanspauwen et al. [79]	Netherlands	196	58.8	1.7	BAL Clara Cell protein 10 ELISA	BAL culture > 10^4^ CFU/mL or > 2% cells with intracellular organisms	AUROCC of 0.586 (0.496–0.676)
Yagmurdur et al. [82]	Turkey	59	71	0.73	ETA culture > 10^4^ CFU/mL	BAL culture > 10^4^ CFU/mL	Sens = 60% Spec = 39%
Zagli et al. [83]	Italy	221	56	2.7	Chest Echography and Procalcitonin Pulmonary Infection Score > 5	ETA culture > 10^4^ CFU/mL	Sens = 80.5% Spec = 85.2%

**Table 3:** Studies comparing index test to a composite reference standard

Reference	Study population				Index test	Reference test	Results (95% confidence intervals where available)
	Country	*n* =	Mean age	M:F ratio			
Charles et al. [53]	France	90	61.1	2.3	Serum PCT (day of first suspicion of clinical infection)	All of (1) new lung infiltrate on the chest X-ray; (2) ETA culture > 10^6^ CFU/mL; (3) CPIS ≥ 6 points; (4) at least 2 SIRS criteria	Sens = 65.2% Spec = 83%
Chen et al. [52]	China	49	54	1.45	Serum PCT serum CRP CPIS	(1) Persistent or new invasive shadows in the lung; (2) At least two below items: temperature more than 38 °C or less than 36 °C; leucocyte count > 10 or < 4 × 10/L; purulent sputum; (3) Any of the item below: bronchoscopic aspiration or sputum specimen bacterial culture +++ or pathogenic bacteria cultured from blood	*CRP* Sens = 68.0% Spec = 58.3% *PCT* Sens = 60.0% Spec = 87.5% *CPIS* Sens = 72.0% Spec = 75.0%
Grover et al. [44]	UK	91	59	1.5	7 marker score: BAL/blood ratio mTREM-1 and mCD11b, BALF sTREM-1, IL-8 and IL-1b, and serum CRP and IL-6	VAP was predefined as CPIS > 5 and positive BALF microbiology. Non-VAP was predefined as CPIS score < 6 and negative microbiology	Sens = 88.9% Spec = 100%
Jovanovic et al. [64]	Serbia	39	47.9	5.5	Serum sCD14-ST PCT CRP Leucocyte count	New persistent pulmonary infiltrates (not otherwise explainable) on CXR > 48 h after admission to the ICU, PLUS one systemic and two pulmonary criteria *Systemic criteria* Fever > 38 °C, white cell count < 4000 WBC/mm^3^ or > 12,000 WBC/mm^3^ altered mental status, with no other recognized cause (for adults older than 70 years of age) *Pulmonary criteria* new onset of purulent sputum (or a change in the character of the sputum, increased respiratory secretions or increased suctioning requirements), worsening gas exchange (desaturations, increased oxygen requirements or increased ventilator demand), new onset or worsening cough, and dyspnoea, tachypnoea, rales or bronchial breath sounds	*sCD14-ST* AUROCC = 0.908 *PCT* AUROCC = 0.863 *CRP* AUROCC = 0.703 *Leucocyte count* AUROCC = 0.668
Klein Klouwenberg et al. [61]	Netherlands	2080	62	1.6	CDC surveillance definition (2013)	Existing local surveillance criteria divided into possible, probable, and definite VAP *Possible* (CPIS > 6, dubious abnormalities on radiographic examination, semi-quantitative culture from respiratory secretions—ETA or bronchoscopic aspirate) *Probable* (CPIS > 6, new or progressive infiltrates, consolidation, cavitation or pleural effusion, BAL culture > 10^4^ CFU/mL or PSB culture > 10^3^ CFU/mL OR positive blood culture with pathogen also isolated from airway culture) *Definite* (CPIS > 6, new or progressive infiltrates, consolidation, cavitation or pleural effusion OR radiographic evidence of lung abscess or empyema, histopathologic evidence of pneumonia (abscess with PMN concentration and positive tissue culture) OR if empyema, positive culture of aspirate))	Sens = 22% (for probable or definite reference standard VAP) Spec = 98%
Klompas et al. [62]	USA	459	55.3	1.5	Objective algorithm of electronic patient record (precursor of CDC surveillance definition 2013)	CDC definition (2008)	Sens = 95 Spec = 100
Liu et al. [68]	China	162	61.6	1.5	BAL neutrophil intracellular organisms	(1) histopathological diagnosis performed within 7 days of bronchoscopy OR (2) BAL culture ≥ 10^4^ CFU/mL, and responded to antibiotic therapy OR (3) rapid cavitation of the lung infiltrate on chest x-ray film or CT scan associated with responded to antibiotic therapy OR (4) positive culture of the pleural effusion, and the same microorganisms isolated from cultures of pleural effusion and lower respiratory tract secretions, and responded to antibiotic therapy OR (5) complete resolution with appropriate antibiotic therapy with no other disease explaining chest radiograph abnormality	Sens = 94.12% Spec = 88.33%
Mauri et al. [71]	Italy	82	59	2.6	BAL fluid pentraxin 3	1. New and persistent radiographic infiltrates associated with at least two of the following: a. Internal body temperature > 38 °C, b. White blood cells count > 12,000 or < 4000 cells/mm^3^ and/or c. Purulent tracheobronchial secretions; AND 2. BAL culture > 10^4^ CFU/mL and/or significant noncontaminant viral load	Sens = 92% Spec = 60%
Medford et al. [72]	UK	150	62.3	1.5	ETA culture > 10^5^ or BAL culture > 10^4^	New/progressive CXR infiltrates without other obvious cause in patients mechanically ventilated for more than 4 days in the ICU and at least 2 of the following: temperature ≥ 38 °C or ≤ 35 °C, white cell count ≥ 12 or ≤ 4 × 10^9^/L, purulent tracheobronchial secretions, with increasing oxygen requirements, computed tomography evidence of a rapidly cavitating infiltrate, positive pleural fluid culture and/or histological evidence of neutrophilic alveolitis, bronchiolitis, and consolidation in conjunction with pleural fluid microbiology, CT evidence, and histological evidence	*BAL* Sens = 64.1% Spec = 83.0% *ETA* Sens = 42.6% Spec = 33.7%
Textoris et al. [78]	France	77	30.75	NR	blood Transcriptome DNA microarray analysis (HuSG9 k)	purulent bronchial sputum; body temperature more than 38 °C or less than 36 °C; leukocytes more than 10 × 10^9^/L or less than 4 × 10^9^/L; chest radiograph showing new or progressive infiltrates; BAL culture ≥ 10^4^ CFU/mL, or ETA culture ≥ 10^6^ CFU/mL	Sens = NR Spec = NR
Vernikos et al. [80]	Greece	54	72	1.3	1) Johansen criteria, 2) Modified CPIS > 6 REF 3) Johansen criteria combined with relative neutrophil count 4) CPIS > 6 combined relative neutrophil count 5) RPDMI^a^ score	CDC definition (2015)	*Johansen criteria* Sens = 85.7% Spec = 73.7% *Modified CPIS > 6* Sens = 62.9% Spec = 73.7% *Johansen criteria combined with relative neutrophil count (20% cut off)* Sens = 67.6% Spec = 81.3% *CPIS > 6 combined relative neutrophil count (20% cut off)* Sens = 47.1% Spec = 81.3% *RPDMI score* Sens = 94.3% Spec = 84.2%
Waltrick et al. [81]	Brazil	168	NR	NR	CDC definition (2013)	CPIS ≥ 7 and miniBAL culture > = 10^4^ CFU/mL	Sens = 37% Spec = 100%

^a^RPDMI score: radiological progression, purulent secretions, duration of mechanical ventilation, immunosuppression

## Reference standard

We did not consider enrolment criteria, including objective criteria for suspicion of VAP, to be part of the reference standard. Out of the 44 included studies, 32 studies (72.7%) compared an index test with a sole microbiological reference standard (see Table 2). One of these studies did not define which of the studied tests were considered index and which were considered reference [54]. Of the remaining 31 studies, culture of BAL fluid was most commonly used as the reference test, forming at least part of the reference standard in 26 (83.8%) studies [40, 41, 43, 45, 48, 49, 51, 55–60, 63, 65–67, 69, 70, 73–77, 79, 82]. BAL culture alone was the sole reference standard in 18 (58.1%) out of the 31 studies [40, 41, 43, 45, 49, 55, 56, 58, 60, 63, 65, 69, 70, 73–76, 82]. Where used, the BAL culture threshold for positivity included > 10 CFU/mL [76], > 10^3^ CFU/mL [56], > 10^4^ CFU/mL [40, 41, 43, 49, 55, 58, 60, 63, 65, 69, 70, 73–75, 82], and > 10^5^ CFU/mL [45]. Three studies using BAL culture > 10^4^ CFU/mL added an additional stipulation on BAL culture results: one requiring correction of microbial growth for plasma to BAL urea ratio [55], one requiring that lavage was bilateral [40], and one specifying culture took place immediately after lavage [60]. BAL culture was used in combination with another criterion regarding assessment of BAL on an additional eight occasions out of the 26 using BAL (30.8%); five out of these eight (62.5%) BAL culture or > 2% lavage cells containing intracellular organisms [48, 66, 67, 77, 79], one out of the eight (12.5%) BAL culture or bronchial washings culture (> 10^5^ CFU/mL) [59], and two out of the eight (11.1%) BAL culture or ETA culture (> 10^6^ CFU/mL [51] or no threshold specified [57]. On the remaining five out of 31 (16.1%) occasions where BAL culture was not incorporated into the microbiological reference standard, three studies used mini-BAL culture > 10^4^ CFU/mL [46, 47, 50], one study used ETA culture > 10^6^ CFU/mL [42]m, and one study used ETA culture > 10^4^ CFU/mL [83], as sole reference standards.

Table 3 summarises the remaining 12/44 (27.2%) studies which compared an index test to a composite reference standard or clinical scoring system [44, 52, 53, 61, 62, 64, 68, 71, 72, 78, 80, 81]. An iteration of the CDC VAP definition was used in two out of the 12 studies (16.7%), one using the 2008 iteration for surveillance of VAP [62] and one using the 2015 iteration for surveillance of VAP (80]. One additional study [64] used a composite reference standard almost identical to the 2008 iteration of the CDC VAP surveillance criteria. The 2008 iteration of the CDC surveillance criteria does not include any microbiological assessment of respiratory samples, and non-culture methods or histopathology may supplant microbiological culture in the 2010 and 2015 iterations of the CDC surveillance criteria [28, 62, 85]. For the remaining nine studies; four out of the nine (44.4%) explicitly incorporated the clinical pulmonary infection score (CPIS) into a wider set of criteria [44, 53, 61, 81]. Seven out nine (77.8%) incorporated radiological assessment [52, 53, 64, 68, 71, 72, 78], eight out of nine (88.9%) incorporated additional clinical signs and symptoms [44, 52, 53, 61, 71, 72, 78, 81] either as part of an existing scoring system such as CPIS or de novo, and all nine studies (100%) incorporated some microbiological assessment into the combined reference standard [44, 52, 53, 64, 68, 71, 72, 78, 81]. In seven of those nine studies (77.8%) positive microbiology was a mandatory criterion for VAP diagnosis [44, 52, 53, 64, 71, 78, 81], and in two of those nine (22.2%), positive microbiological cultures were an optional criterion for VAP diagnosis [68, 72]. Amongst all included studies microbiological assessment did not form any part of the mandatory or optional criteria for VAP diagnosis in only two studies [62, 64], diagnosis of VAP included an optional microbiological assessment component in another two studies (i.e. VAP diagnosis *could* be made without recourse to microbiological assessment) [68, 72], and in 39 studies microbiological assessment was the sole or a mandatory component [40–53, 55–60, 63–71, 73–79, 81–83]. Four studies incorporated an optional histopathologic element into the reference standard [61, 68, 72, 80], but it was mandatory in none.

Out of all 44 included studies, 37 (84%) incorporate BAL in either the reference standard or index test [40–51, 54–60, 63, 65–79, 81, 82]. Twenty two studies (50%) included a precise lavage procedure in the methodology [40, 41, 43, 45, 48, 51, 55–58, 60, 63, 66–69, 71, 72, 74–76, 82]. Of these, 14 described an initial discard of aspirated fluid, considered to be uninformative bronchial fluid [40, 41, 43, 51, 57, 58, 60, 63, 68, 69, 71, 72, 74, 82]. The median volume of instilled fluid used to generate the discarded fluid was 20 mL (range 20 mL to 50 mL). The median total volume of instilled fluid (including that intended for discard) was 150 mL (range 80 mL to 200 mL).

## Discussion

To the best of our knowledge, this is the first and most comprehensive systematic review aiming to evaluate the reference standard tests used to evaluate novel index tests for suspected VAP since the publication of Rea-Neto and colleagues systematic review of diagnostic methods in 2008 [32]. We reviewed papers comparing a novel index test against a chosen reference standard to identify what reference standards have been used in diagnostic evaluation research for VAP. To deliver a high-quality systematic review, we excluded papers with a high risk of bias and all papers included in this review fulfil at least 5 out of the 8 criteria we included from the CASP checklist.

The microbiological culture was the sole or a component criterion in the vast majority of studies. Overall, the culture of BAL fluid was the most common reference standard, with the most common growth threshold being > 10^4^ CFU/mL. This was occasionally used in combination with another reference standard, such as the demonstration of BAL cells with intracellular organisms exceeding 2% of the total number of cells. Composite reference standards incorporating a variety of existing clinical scores, existing surveillance definitions, radiological assessments, clinical parameters, and microbiological methods including culture were used in the remaining studies. A large variation in practice, processes, and reference standards were detected, highlighting the inconsistency in the current diagnosis of VAP and making a meta-analysis of diagnostic accuracy challenging. Biological, clinical, and statistical heterogeneity makes comparisons across the different studies difficult and subjective. We display a variable and generally good quality of the papers, and the review provides an indication of what has been and is being done in this area globally with respect to the use of reference standard in the diagnostics of VAP. The line between composite criteria and a sole microbiological criterion was often blurred. Many studies in the sole microbiological criterion group had strict objective clinical and radiological enrolment criteria. Where these criteria are applied pre-enrolment and therefore applied to both index tests and reference standards we have not incorporated them into a description of the reference standard.

A key question in diagnostic accuracy research when reference standards are imperfect is whether the reference standard used to assess novel diagnostics should be ‘more inclusive’ (higher sensitivity, lower specificity) or ‘less inclusive’ (lower sensitivity, higher specificity). Using microbiological criteria alone exhibits good face validity but risks missing cases of ‘true VAP’ or including false positives through contamination (although prior specification of clinically suspected VAP reduces this risk). Importantly, both possibilities are potentially strongly influenced by operator technique/expertise, especially for BAL; this contrasts with diagnostics reliant on blood sampling or imaging. BAL culture was the most common microbiological method found in this review. The use of BAL culture is potentially problematic for several reasons. Firstly, in a recent systematic review, when compared to the reference standard of histopathological examination of lung tissue, BAL culture had a sensitivity of 71.1% and specificity of 79.6% [86] echoing previous findings that microbiological examination does not correlate well with histopathological examination [32]. Secondly, the timing and nature of prior antibiotic therapy may adversely affect sample positivity [87, 88], although this problem is conceivably solved by incorporating a criterion addressing percentage of host cells containing invading organisms, a measure not affected by prior antibiotic therapy [75]. Thirdly, the BAL procedure itself is not standardised, and the requirements for sample collection are not uniform. Whilst this may have little impact on bacterial growth, a fact confirmed by one of the included studies [55], the variety of studies that utilised bronchial discard may plausibly lead to a variety in sensitivity at detecting the causative pathogenic organism. Sole microbiological criteria also risk introducing cases of ‘false VAP’ through contamination [87], although this risk is reduced by using distal or protected specimens. Of relevance, the quality and consistency of BAL procedures are likely to be higher in studies than during routine clinical practice, which could further influence its validity.

Using composite criteria may conceivably address the problem of missing cases of ‘true VAP’, and the number or thresholds of additional criteria is not limited. Additional criteria can be made mandatory to increase specificity or made optional to increase sensitivity. Some studies in this review rely on existing surveillance definitions for VAP or use their own composite standards. The existing surveillance definitions were designed to objectively and reproducibly monitor VAP rates not to identify true VAP in a robustly sensitive and specific manner, although as a quality indicator face validity amongst clinicians is important. Other composite studies incorporated radiological assessments into the reference standard. It has been shown that chest x-ray changes are not considered integral to the diagnosis by many clinicians [89], that the performance characteristics of chest x-ray may not meet the requirements as a diagnostic standard [90–92], and that inter- and intra-observer variability is high in chest x-ray assessment [93, 94]. These issues mean that incorporation of radiology into any novel reference standard should be undertaken with caution. Many studies incorporate clinical signs which plausibly reduces the risk of false positives, and although this makes physiological sense there is minimal evidence to support this. Klompas et al. showed, in the development of the novel CDC VAP surveillance algorithm, that deterioration in oxygenation after a period of stability was associated with clinically important outcomes but the addition of other clinical measures such as abnormal temperature, abnormal white blood cell count, or purulent secretions was not [95]. However, a lack of correlation with clinically important outcomes is not the same as a lack of correlation with a true diagnosis of VAP; this issue is relevant when the decision based on the test relates to a therapy (antibiotic use) rather than prognosis.

No studies relied upon histopathological diagnosis of VAP to confirm the diagnosis. This is not surprising for practical reasons: it cannot be routinely and safely undertaken in all patients with suspected VAP either at the time of the index test or later. Histopathological analysis may also be inaccurate due to sampling artefacts, the lack of representation of a small piece of tissue, and displacement in time from the period of peak infection. It is not possible to provide certainty about the appropriate reference standard in diagnostic evaluation research for VAP following this systematic review, which simply identifies the methods chosen by researchers and confirms the lack of a standardised approach. Researchers must decide whether it is more important to be ‘more inclusive’ or ‘less inclusive’, and future comparisons may wish to employ the strategy deployed by one of the studies in this review [61]: using a graded certainty of VAP from possible to probable to definite using a composite definition.

There are three main limitations to our review. Firstly, in order to be diagnosed with VAP, a patient must be at risk of VAP, and there is no standard definition for patients at risk. For the purposes of this study, we defined those at risk of VAP as those who have undergone more than 48 h of mechanical ventilation. Secondly, many included studies enrolled only patients with suspected VAP, and this means many listed reference standards must be prefixed with “clinically suspected VAP”. This level of clinical suspicion was not systematically collected by us. This is particularly noteworthy in considering the reference standards listed in Table 3. Thirdly, although data extraction for this review was completed before the impact of Coronavirus Disease 2019 (COVID-19), the pandemic nonetheless interfered with the delivery time of this review.

## Conclusion

BAL culture with a microbiological growth threshold of > 10^4^ CFU/mL is the commonest reference standard used to examine the utility of a novel index test for VAP amongst patients who are at risk for and clinically suspected of VAP. Composite reference standards were used in approximately 25% of reviewed studies. Nearly all reference standards for VAP identified in this review required some microbiological confirmation of infection. The studies identified in this review highlight the need for a standardised approach to diagnosis VAP which may include the development of a data-driven composite reference standard from large cohort studies.

## Supplementary Information

**Additional file 1.** MEDLINE search example.

**Additional file 2.** Quality assessment and data extraction form.

## Data Availability

The datasets used and/or analysed during the current study are available from the corresponding author on reasonable request.

## Abbreviations

BAL: Broncho-alveolar lavage
CDC: Centers for disease control and prevention
CC-10: Clara cell protein 10
COVID-19: Coronavirus Disease 2019
CASP: Critical appraisal skills programme
CBA: Cytometric bead array
ETA: Endotracheal aspirates
ECDC: European centre for disease prevention and control
GC-tof-MS: Gas chromatography time-of-flight mass spectrometry
GS: Gram’s stain
ITU: Intensive therapy unite
LUS: Lung ultrasound
MeSH: Medical subject headings
CPIS: Modified clinical pulmonary infection score
NHSN/CDC: National healthcare safety network/Center for disease control and prevention
PCR: Polymerase chain reaction
PSB: Protected specimen brush
SQ: Semi quantitative
PCT: Serum procalcitonin
sTREM-1: Type 1 soluble triggering receptor expressed on myeloid cells
U/S: Ultrasound
VAE: Ventilator-associated events
VAP: Ventilator-associated pneumonia
VOCs: Volatile organic compounds
