# Supplementary material for: Systematic review of studies investigating ventilator associated pneumonia diagnostics in intensive care
Source: BMC Pulm Med. 2021 Jun 9;21:196. doi: 10.1186/s12890-021-01560-0 (PMC8189711; doi:10.1186/s12890-021-01560-0)
Supplement: Supplementary file 1 — Additional file 1. MEDLINE search example. [file 12890_2021_1560_MOESM1_ESM.pdf]

Appendix 1: MEDLINE search example.

Search	Query	Items found
#4	Search **(((((((((((((((((((((((((((("chest x-ray") OR "chest x?ray") OR “chest roentgenography”) OR “chest roentgenogram”) OR “chest radiograph”) OR “chest radiography”) OR “chest radiogram”) OR “lung ultrasound”) OR “lung ultrasonography”) OR “lung sonograph”) OR “lung echograph”) OR “lung ultrasonics”) OR “lung biopsy”) OR bronchoscop*) OR “bronchoalveolar lavage”) OR “protected specimen brush”) OR diagnostic*) OR “endotracheal aspiration”) OR “chest ultrasound”) OR biomarker*) OR “biological marker*”) AND ("2008/01/01"[PDat]))) OR ((((((((((((((((((((((((("chest x-ray")[MeSH Terms] OR "chest x?ray")[MeSH Terms] OR “chest roentgenography”)[MeSH Terms] OR “chest roentgenogram”)[MeSH Terms] OR “chest radiograph”)[MeSH Terms] OR “chest radiography”)[MeSH Terms] OR “chest radiogram”)[MeSH Terms] OR “lung ultrasound”)[MeSH Terms] OR “lung ultrasonography”)[MeSH Terms] OR “lung sonograph”)[MeSH Terms] OR “lung echograph”)[MeSH Terms] OR “lung ultrasonics”)[MeSH Terms] OR “lung biopsy”)[MeSH Terms] OR bronchoscop*)[MeSH Terms] OR “bronchoalveolar lavage”)[MeSH Terms] OR “protected specimen brush”)[MeSH Terms] OR diagnostic*)[MeSH Terms] OR “endotracheal aspiration”)[MeSH Terms] OR “chest ultrasound”)[MeSH Terms] OR biomarker*)[MeSH Terms] OR “biological marker*”[MeSH Terms])) AND ("2008/01/01"[PDat]))) AND ("2008/01/01"[PDat]))) AND (((((“ventilator associated pneumonia”) OR "ventilator-associated-pneumonia") OR "ventilator-associated-pneumonia"[MeSH Terms]) OR “ventilator associated pneumonia”[MeSH Terms]) AND ("2008/01/01"[PDat]))) AND ((((((((((((((((“intensive care unit*”) OR “intensive-care-unit*”) OR “intensive treatment unit*”) OR “intensive-treatment-unit*”) OR “intensive-therapy-unit*”) OR “intensive therapy unit*”) OR “high-dependency-unit*”) OR “high dependency unit*”) OR “intensive care unit*”[MeSH Terms]) OR “intensive-care-unit*”[MeSH Terms]) OR “intensive treatment unit*”[MeSH Terms]) OR “intensive-treatment-unit*”[MeSH Terms]) OR “intensive-therapy-unit*”[MeSH Terms]) OR “intensive therapy unit*”[MeSH Terms]) OR “high-dependency-unit*”[MeSH Terms]) OR “high dependency unit*”[MeSH Terms]) AND ("2008/01/01"[PDat]))** Filters: **Publication date from 2008/01/01**	3100
#3	Search **(((((((((((((((“intensive care unit*”) OR “intensive-care-unit*”) OR “intensive treatment unit*”) OR “intensive-treatment-unit*”) OR “intensive-therapy-unit*”) OR “intensive therapy unit*”) OR “high-dependency-unit*”) OR “high dependency unit*”) OR “intensive care unit*”[MeSH Terms]) OR “intensive-care-unit*”[MeSH Terms]) OR “intensive treatment unit*”[MeSH Terms]) OR “intensive-treatment-unit*”[MeSH Terms]) OR “intensive-therapy-unit*”[MeSH Terms]) OR “intensive therapy unit*”[MeSH Terms]) OR “high-dependency-unit*”[MeSH Terms]) OR “high dependency unit*”[MeSH Terms]** Filters: **Publication date from 2008/01/01**	100268
#2	Search **(((“ventilator associated pneumonia”) OR "ventilator-associated-pneumonia") OR "ventilator-associated-pneumonia"[MeSH Terms]) OR “ventilator associated pneumonia” [MeSH Terms]** Filters: **Publication date from 2008/01/01**	7914

Search	Query	Items found
#1	Search **(((((((((((((((((((((("chest x-ray") OR "chest x?ray") OR “chest roentgenography”) OR “chest roentgenogram”) OR “chest radiograph”) OR “chest radiography”) OR “chest radiogram”) OR “lung ultrasound”) OR “lung ultrasonography”) OR “lung sonograph”) OR “lung echograph”) OR “lung ultrasonics”) OR “lung biopsy”) OR bronchoscop*) OR “bronchoalveolar lavage”) OR “protected specimen brush”) OR diagnostic*) OR “endotracheal aspiration”) OR “chest ultrasound”) OR biomarker*) OR “biological marker*”) AND ("2008/01/01"[PDat]))) OR (((((((((((((((((((((("chest x-ray")[MeSH Terms] OR "chest x?ray")[MeSH Terms] OR “chest roentgenography”)[MeSH Terms] OR “chest roentgenogram”)[MeSH Terms] OR “chest radiograph”)[MeSH Terms] OR “chest radiography”)[MeSH Terms] OR “chest radiogram”)[MeSH Terms] OR “lung ultrasound”)[MeSH Terms] OR “lung ultrasonography”)[MeSH Terms] OR “lung sonograph”)[MeSH Terms] OR “lung echograph”)[MeSH Terms] OR “lung ultrasonics”)[MeSH Terms] OR “lung biopsy”)[MeSH Terms] OR bronchoscop*)[MeSH Terms] OR “bronchoalveolar lavage”)[MeSH Terms] OR “protected specimen brush”)[MeSH Terms] OR diagnostic*)[MeSH Terms] OR “endotracheal aspiration”)[MeSH Terms] OR “chest ultrasound”)[MeSH Terms] OR biomarker*)[MeSH Terms] OR “biological marker*”[MeSH Terms])) AND ("2008/01/01"[PDat]))** Filters: **Publication date from 2008/01/01**	985870
