# Supplementary material for: Systematic review of studies investigating ventilator associated pneumonia diagnostics in intensive care
Source: BMC Pulm Med. 2021 Jun 9;21:196. doi: 10.1186/s12890-021-01560-0 (PMC8189711; doi:10.1186/s12890-021-01560-0)
Supplement: Supplementary file 2 — Additional file 2. Quality assessment and data extraction form. [file 12890_2021_1560_MOESM2_ESM.pdf]

Appendix 2: Quality assessment and data extraction form

<u>Reviewer Name</u>:

<u>Date of the review</u>:

General information

Study title	
Authors	
Year of publication	
Publishing journal	
Country of study	
Study funding/conflict of interest	

Eligibility

1. Is this a case-controlled study?

YES ☐ If YES, *please exclude and return to Basem immediately*

NO ☐ If NO, please continue

2. Was there a clear question for the study to address?

Consider: A question should include information about the population, the test, the setting, and the outcomes

YES ☐ If YES, please continue

NO ☐ If NO, *please exclude and return to Basem immediately*

3. Was there a comparison with an appropriate reference standard?

Consider: Is this reference test(s) the best available indicator in the circumstances?

YES ☐ If YES, please continue

NO ☐ If NO, *please exclude and return to Basem immediately*

4. Did all patients get the diagnostic test and reference standard?

Consider: Were both received regardless of the results of the test of interest? Check the 2x2 table (verification bias)

YES ☐ NO ☐

5. Could the results of the test have been influenced by the results of the reference standard?

Consider: Was there blinding? were the tests performed independently? review bias.

YES ☐ NO ☐

6. Is VAP status of the tested population clearly described?

Consider: Presenting symptoms. VAP stage of severity. Co-morbidity. Differential diagnoses (spectrum bias)

YES ☐ NO ☐

7. Were the methods for performing the test described in sufficient detail?
Consider: Was a protocol followed?

YES ☐ NO ☐

8. Were the results clearly presented?
Consider: Are the sensitivity and specificity and/or likelihood ratios presented? Are the results presented in such a way that we can work them out?

YES ☐ NO ☐

9. How sure are we about the results?
Consider: Could they have occurred by chance? Are there confidence limits? What are they?

YES ☐ NO ☐

Methods:

Research method	
Population of interest	
Inclusion criteria	
Exclusion criteria	

Population and Sampling

Population	
Sample size (enrolled)	
Sample source	
Sampling method	
Participants age	
Participants Sex (M/F)	
Number of participants who completed the study	

Methods

Study main aim	
Primary outcome	
Secondary outcome	
Index test(s) and Synonyms	
Reference standard (comparator test)	
Description of procedure	
Clinical expertise or training required for the procedure	
Equipment required for the test	
Indicated results range for action (what are the thresholds that triggers action?)	

Results

Study results	Accuracy	
	Effectiveness	
	Clinical acceptability	
Reported statistical results		
Any adverse effects of the procedure		
Treatment decisions	1. No action<br>2. Initiated<br>3. Amended<br>4. Stopped	
Conclusion		
Reported strengths of test		
Reported weaknesses of test		
Additional comments		
